# Identified gamification opportunities for digital patient journey solution during an arthroplasty journey: secondary analysis of patients' interviews

**DOI:** 10.1002/nop2.1215

**Published:** 2022-04-23

**Authors:** Johanna Jansson, Elina Laukka, Outi Kanste, Jonna Koivisto, Miia Jansson

**Affiliations:** ^1^ 6370 Research Unit of Nursing Science and Health Management University of Oulu Oulu Finland; ^2^ 7840 Faculty of Information Technology and Communication Sciences Tampere University Tampere Finland; ^3^ 6370 Research Group of Medical Imaging, Physics and Technology University of Oulu Oulu Finland

**Keywords:** arthroplasty, gamification, patient journey

## Abstract

**Aim:**

The use of gameful design for supporting health‐related behaviours has been one of the major trends in health technology. An opportunity to increase engagement and motivation in a given health behaviour and the possibility of reaching improved outcomes through continued or consistent behaviour could be provided by gamification. This study aimed to identify gamification opportunities for digital patient journey solutions to increase patients' engagement and motivation for health‐related behaviour during an arthroplasty journey.

**Design:**

A secondary analysis.

**Method:**

Semistructured interviews were performed among 20 elective primary total hip and knee arthroplasty patients in a single joint‐replacement centre in Finland during autumn 2018. NVivo software was used for deductive content analysis. The study was conducted among 20 patients in a single joint replacement centre during 2018.

**Results:**

Several opportunities for gamification were identified for digital patient journey solutions, which could be used in advanced care to increase patients' engagement and motivation for health‐related behaviour during the arthroplasty journey. These opportunities were identified related to five dimensions: accomplishment, challenge, guided, playfulness and social experience. Clear, scheduled, progressive and personalized goals with an activity tracking, real‐time timespan visualization and social networking with peers, support networks and healthcare providers could be provided. Opportunities for competition and immersion were not identified.

## 1 INTRODUCTION

The demand and costs for a total hip arthroplasty (THA) and total knee arthroplasty (TKA) will be rising continuously and significantly over the next decades (Ackerman et al., 2019; Australian Institute of Health & Welfare, 2020; Pabinger et al., 2018) due to ageing population demographics and obesity (Culliford et al., 2015). At the same time, the average length of hospital stay has shortened by 1 day due to fast‐tracking (Williams et al., 2015; Wolford et al., 2015), changing the patient's role from passive receiver of care to active participant and a co‐producer of healthcare services (Vieresjoki et al., 2021). This warrants action to develop patient‐centred services in a cost‐effective and efficacious way.

Digitalization provides great potential for improving patient‐centred services in a cost‐effective way, while successful well‐being aware and user‐centred digital design requires an understanding of the desired change and how technology should work, and that the change is possible (Laukka et al., 2020; Vieresjoki et al., 2021). In recent years, the application of game‐like elements (including components, mechanisms and dynamics) into non‐gaming contexts, such as rehabilitation, has gained popularity in the health field. This design approach has often been titled as gamification. Research trends is gamification represents a new and innovative approach which is likely to develop in the near future also thanks to technological innovations and artificial intelligence. In fact, the global healthcare gamification market is supposed to grow from $1.1 billion in 2020 to $8.6 billion by 2027 (Healthcare Gamification Market Research Report, 2022).

Gamification can provide an opportunity to increase patient engagement and motivation in a given health behaviour and the possibility of reaching improved outcomes through continued or consistent behaviour (Jansson et al., 2020). Instead of exergames, the focus should also be paid on remote monitoring, patient counselling and social support (Fernandes et al., 2021). To gain this understanding, continuous cooperation with patients and healthcare providers is needed (Gjellebæk et al., 2020). In addition, studies have shown that both healthcare professionals and leaders seem to support digital services if they provide benefits for the patients (Kujala et al., 2018; Laukka et al., 2021). Thus, understanding patients' views of the opportunities of the digital services, such as gaming solutions, may also increase healthcare professionals' and leaders' support for their usage.

## 2 BACKGROUND

During the past years, the use of gameful design for supporting health‐related behaviours has been one of the major trends in health technology (Hamari & Koivisto, 2015). Providing similar experiences as games do in contexts that are not commonly considered to be game‐like and, at the same time, motivating the user of the gamified system towards certain behaviour, are the goals of the design approach of gamification (Huotari & Hamari, 2016). These experiences evoked by games have often been referred to as gameful experiences, whose definition includes seven dimensions: accomplishment, challenge, competition, guided, immersion, playfulness and social experience (Högberg et al., 2019).

The need to experience achievement and the sense of progress in reaching set goals related to the behaviour targeted with the gamification is in relationship with accomplishment dimension while challenge dimension is related to the experience of being challenged with the gamified behaviour, and the behaviour requires increasing effort (Högberg et al., 2019). Having specific and challenging goals increases related task performance also according to goal‐setting theory (Locke & Latham, 2002). Prior research on creating experience of accomplishment has indicated that clear tasks and goals are some of the most common elements and affordances implemented in gamified systems, while progress bars are used to indicate the completion of provided tasks or reaching goals (Koivisto & Hamari, 2019). The experiences of being guided, related to tasks and progress in relationship with the target behaviour or activities are related to a guided dimension, while a social experience dimension is related to the experiences arising from the perceived social presence of other real or virtual individuals (Högberg et al., 2019).

In the context of the arthroplasty journey, gamification and game‐based solutions can provide various possibilities for personalized counselling, monitoring and social support (Jansson, Koivisto, et al., 2020) in order to increase the physical and cognitive performance of THA/TKA patients (Brem et al., 2010; Fung et al., 2012; Lehrl et al., 2012; Ling et al., 2017). However, the use of gameful solutions is not yet common, and research in the area is scarce (Fernandes et al., 2021). In addition, due to the contextuality of gameful solutions and their design, specific needs of different user groups and the consequential challenges in applying results from one context to another, further research in different healthcare settings is warranted.

Therefore, this study aims to identify gamification opportunities for digital patient journey solutions to increase patients’ engagement and motivation for health‐related behaviour during an arthroplasty journey. The study is part of a larger research project that develops, implements and evaluates thespi‐global\ effectiveness of a digital patient journey solution together with patients, healthcare providers, technology providers and researchers. The research question is as follows:

What opportunities for inducing the gameful experience dimensions of accomplishment, challenge, competition, guided, immersion, playfulness and social experience can be identified from the TKA and THA patients’ interviews?

## 3 METHODS

### 3.1 Design

A secondary analysis of qualitative data collected in 2018 is reported in this paper. The original study was exploring patient satisfaction and experiences during elective, primary fast‐track THA/TKA journey (Jansson et al., 2020).

### 3.2 Setting and participants

The selection of patients for the primary study was done by using convenience sampling (Polit & Beck, 2017). Patients were approached by the research nurse, and they were recruited during (a) a preoperative surgical visit; (b) a surgical visit; and (c) a post‐operative outpatient visit. For a patient to be included in the study, they had to (1) be ≥18 year; (2) be able to speak, read and understand Finnish; (3) be undergoing, or had already undergone, elective primary THA/TKA; and (4) have a smartphone or tablet computer. Patients with rheumatoid arthritis (RA) and revision surgery were excluded because the aetiology and treatment of RA and THA/TKA revision differ from osteoarthritis and primary THA/TKA.

### 3.3 Data collection

Semistructured interviews were conducted among 20 TKA and THA patients in a single joint replacement centre in a 900‐bed, tertiary‐level university teaching hospital in Finland during autumn 2018. Interviews were carried out at the hospital by a qualitative methodologist (PhD), and it was made clear to patients that the researcher (female) was not a member of the clinical staff. The semistructured interviews, with more specific questions in cases of brief answers by a patient or a need to hear more by interviewer, were conducted. To evaluate the level of the patients' gaming experience and their playing behaviour, the following questions were asked: ‘Do you play games?’ and ‘Which games and how often?’. To evaluate the level of the patients’ personal playing orientation related to the importance of orientations of achievement, immersion and socializing, the following question was asked: ‘How important are different game elements for you?’. The mean length of the interview was 36.8 min, and the total length of all interviews was 12 hr and 16 min (Jansson, Harjumaa, et al., 2020). As the data were saturated, the sample size was considered to be sufficient.

### 3.4 Data analysis

Secondary analysis of qualitative data was used to perform additional analyses on original data set and to apply a new perspective to the original data (Tate & Happ, 2018). The secondary analysis reported in this paper identified gamification opportunities for digital patient journey solutions. Additionally, the analysis evaluated the level of gaming experience, playing behaviour and playing orientation.

During the data collection, the interviews were audio recorded, and the recordings were transcribed verbatim by a transcription service provider to ensure a complete and accurate recording of each response. Furthermore, the transcripts were pseudonymized for the analyses. A deductive content analysis approach was used to analyse the collected data (Kyngäs et al., 2019). The predetermined categorization matrix, including main, generic and subcategories, was used (Högberg et al., 2019; Jansson, Koivisto, et al., 2020). First, the data were coded according to the main categories of framework dimensions (Högberg et al., 2019). As a second phase, the data were coded according to generic and subcategories (Jansson, Koivisto, et al., 2020). First, the initial coding of the collected data was conducted by the first author (JJ). Then, the initial coding was reviewed by a PhD‐qualified author (MJ) to ensure that all adequate information was coded. NVivo software was used for data analysing (QSR International Pty Ltd., release 1.5.1).

### 3.5 Rigour

In this study, the trustworthiness was evaluated by using the criteria for reliability of qualitative research (Kyngäs et al., 2019; Lincoln & Guba, 1985; Shenton, 2004). The criterion of credibility was achieved through audio recorded transcriptions, and data were respected as such during the analysis and reporting phase of the study. The methodology of the study was described in detail, and the study results were reported without commentary to ensure the transferability of the findings. The criterion of dependability was ensured through an audit trail. The initial coding of the interview data conducted by the first author (JJ) was reviewed by the PhD‐qualified author (MJ) to ensure that all adequate information was coded. Confirmability was ensured by receiving feedback from the other research members in the team, who provided alternative perspectives. In order to establish authenticity, excerpts from the interviews in their original form are included in the study report. The reporting of the findings of the study followed the COREQ checklist (Tong et al., 2007) (See Appendix S1).

### 3.6 Ethical starting points

The primary study (Jansson, Harjumaa, et al., 2020) was reviewed by a local ethics committee (Decision No: 83/2018). The aim of the study was explained to the patients, and they were also informed that interviews were audio recorded and the collected data would be transcribed and pseudonymized. A signed informed consent was collected from patients prior to inclusion in the study to ensure that the participation was voluntary (World Medical Association Declaration of Helsinki, 2013). A data processing agreement has been signed by all researchers processing the raw interview data.

## 4 RESULTS

### 4.1 Demographics

Participants consist of 20 patients, who were undergoing or had undergone elective primary THA/TKA. Of the total 20 patients, nine patients with THA (45.0%) and 11 with TKA (55.0%) were included. The ages of the nine THA patients [seven women (77.8%) and two men (22.2%)] ranged from 52–74 years, while the mean age was 66.2 and *SD* 7.8. The ages of 11 TKA patients [six women (54.5%) and five men (45.5%)] ranged from 56–76 years, while the mean age was 68.1 and *SD* 6.6 (*N* = 20).

#### 4.1.1 Playing behaviour

Of the total 20 interviewees, some types of digital games (e.g. solitaire, puzzles and word games) were reported to be played by six interviewees, either on a smartphone, a tablet or a PC. The frequency of playing such games varied from daily play to playing rarely. One interviewee reported also playing daily other types of casual mobile games and console games. Non‐digital games (e.g. board games, sudoku, crossword puzzles and playing cards) were reported to be played by 12 interviewees. The frequency of non‐digital game play varied from daily play to playing weekly or rarely. Furthermore, seven interviewees reported playing gambling games (e.g. lotteries and sports betting) or buying scratch cards. The frequency of participating in gambling games varied from weekly to rarely. Three interviewees reported that they did not play any kind of games.

#### 4.1.2 Playing orientation

Four interviewees reported some level of achievement orientation regarding their playing behaviour. Half of the interviewees highlighted it as extremely important, and the other half considered it somewhat important. Competition and immersion as a playing orientation were not mentioned by the interviewees. The social aspects of playing were considered as important by four interviewees. All other interviewees considered the socialness of playing to be only somewhat or very slightly important.

#### 4.1.3 eHealth behaviour and orientation related to a digital patient journey solution

Of the total 20 interviewees, seven reported that they did not use any kind of eHealth services. Some types of eHealth services (e.g. National Kanta services, Oulu's Omahoito‐online service) were reported to be used by 13 interviewees to, for example, renewal prescription, make appointments and monitor their own health information. Eleven interviewees initially exhibited a negative attitude towards digital patient journey solutions. The following quote from the interviews illustrates this view: ‘Guidance has become too digital. That is because a lot of people are older. They don't have all of these, they don't know how to use them, they're helpless’ (ID207, female with TKA). Later, four of these 11 interviewees showed positive attitudes towards this topic when mentioned by the interviewer. The following quote from the interviews illustrates this view: ‘I'd probably use it if I knew how to use it’ (ID207, female with TKA). Of the total 20 interviewees, nine interviewees showed a positive attitude towards a digital patient journey solution from the start. The following quote from the interviews illustrates their view of a digital patient journey solution's benefits: ‘it's such a smooth way of handling the whole package from start to finish. That they're not in pieces over there and in pieces here or on paper or something else’ (ID213, female with TKA).

### 4.2 Identified gamification opportunities

According to Högberg et al. (2019), gamification opportunities were identified on five dimensions: accomplishment, challenge, guided, playfulness and social experience. These issues reflected patients’ points of view during the arthroplasty journey. The open codes for various game elements and affordance opportunities for inducing the gameful experience dimensions within each category are presented in Table 1.

**TABLE 1:** Examples of identified gamification opportunities

Main category	Generic category	Subcategory	Description
Accomplishment	Clear goals		Pre‐ and post‐operative goals could be provided via a logical chronological checklist, which includes all required actions to be done by the patient
		Daily goals	Daily goals could be provided in relation to pre‐ and post‐operative tasks
		Weekly goals	Weekly goals could be provided in relation to overall medication and pre‐operative weight management
		Discharge goals	Discharge goals could be provided pre‐operatively in relation to familiarize the patient with the discharge criteria, to motivate the patient in their new active role, to give better accessibility to monitor the fulfilment of the discharge criteria independently
		Long‐term goals	Long‐term goals could be provided pre‐operatively related to progressive mobility, dietary changes, weight management and smoking cessation. Post‐operatively long‐term goals could provide progressive rehabilitation exercises
	Activity tracking		Activity tracking could provide guidance and monitoring with reminders related to pre‐ and post‐operative goals to motivate patients. It could be offered related to rehabilitation exercises, stretch training, distance, pace, heart rate, energy consumption, creating a sense of security, motivating and monitoring your own development (compared to average)
		Diary	Diary with reminders (tick the box) could provide an opportunity to report, monitor and get feedback related to exercising, weight, pulse and blood pressure and food diary
		Check lists	Chronological and detailed (e.g. each aid device must have its own checkbox) check list (tick/untick the box) could include all required actions to be done by patient
		Notifications and reminders	Chronological and clear reminders (e.g. SMS, email) could be provided related to all required actions to be done by the patient. Notifications could be provided related to events in the THA/TKA surgery journey. Reminders could support in reaching clear goals and notifications (e.g. notification of arrival of missing aid) could enable to keep the patient informed. Pre‐operative activity tracking could provide guidance and reminders related to muscle fitness training, checking skin conditions, undergoing dental examinations, acquiring and use of aids and discontinuance of natural product medications. Post‐operatively, activity monitoring could provide guidance and reminders related to the safe discontinuance of pain medication and the physiotherapist visit of the health centre
Challenge	Adaptive or increasing difficulty or upward tendency		Adaptive/increasing/upward tendency in repetition and duration of exercises could be provided to support the patient's better recovery from surgery and the patient's safe and progressive exercising
	Personalized challenge		Challenges and exercises must be personalized based on the patient's background factors (e.g. activity of exercising, potential problems and case of both hip surgeries), framework of the living environment (e.g. single people, drivers, travellers in car) and personal target of post‐operative mobility (e.g. description of work, active exerciser)
Guided	Content of counselling		Content of counselling could include information about health guarantee, health behaviour, preparation for surgery, the surgery itself, rehabilitation, traveling (e.g. ordering of taxi at time of discharge, traveling in a car, driving a car), medication (e.g. injecting medicine and discontinuance of pain medication), complications, digital joint certificate and contact information
		Information input and provision	Information input and provision could enable collecting data from patients
		Sensors	Information input and provision could enable input and provision information from sensors (e.g. kinetic and pulse sensors) and enable remote monitoring

	Implementation of counselling	Methods of counselling	Implementation of counselling could provide methods for digital counselling by enabling digital counselling material (including video clips, check list and diary), remote visits with the help of a video call, a message system (incl. chat, email), a social networking platform (including health professionals, peers and support network), a feedback provider (e.g. results reported by patient via diary) and navigator (e.g. for visits in and inside hospital) could be provided
		Personalized counselling	Personalized counselling, with content that is meaningful for patients with THA or TKA, could be provided to patients to address, e.g. fears, Certain lifestyles (e.g. workers, active/inactive exercisers), personal needs (e.g. no consent to transfusion, previous unsuccessful TKA/THA), special needs (e.g. urgent need for surgery), overall needs (e.g. balancing of other illnesses), surgery time during winter (e.g. safe exercising on slippery surfaces) and distant municipality of residence (e.g. to enable smooth visits to hospital)
		Patient‐centeredness	Needs‐based facilities (e.g. room size in inpatient ward, bed height set according to height of patient and needed aids available), needs‐based care (needs‐based support (e.g. for getting out of bed for the first time, early mobilization) and needs‐based diet (e.g. for diabetic))
	Benefits of counselling	Engagement	Benefits of counselling could provide patient' s engagement related to life‐style change, fulfil discharge criteria and rehabilitation exercising by providing clear guidance and by tracking activity in reaching clear personalized goals
	Performance feedback		A performance feedback channel for patients could be provided and acknowledgement of the reception of feedback
Warnings		Warnings could be sent to patients if needed (e.g. analgesic discharge has not been started)
Playfulness	Ease of use		Ease of use could be provided with the help of a simple and clear user interface (e.g. patient could easily input information related to medication and book appointments) and long‐lasting battery in sensors
	Visualization		Visualization could be provided related to status of rehabilitation
		Timeline	Real‐time timeline could be visualized to increase transparency of the entire TKA/THA patient journey and to ensure that there are no gaps in the information flow between different service providers (e.g. referral from private and occupational health providers)
Social experience	Encouragement		Encouragement could be provided related to fears, lifestyle changes, discharge and rehabilitation
	Monitoring		The ability to monitor feedback provided by health professionals could be provided
		Accountability	Patient accountability related to remaining actions could be increased by providing the ability for a patient to monitor remaining actions in relationship with reaching clear goals
		Visualization	Possibility to monitor visualized feedback (e.g. traffic lights feedback of action's status related to reaching clear goals reported by the patient) and reports (e.g. epicrisis, laboratory results) could be provided
	Social networking		Social networking could be provided via a platform
		Peer support	Peer support could be provided to enable peer support from other patients
		Support network	Social networking could be enabled to the support network (e.g. family) to enable information flow (e.g. real‐time timeline of patient's TKA/THA journey, navigator, guidance related to homecare)
		Professional support	Professional support could be provided for direct communication (e.g. chat and video call) with healthcare providers and via a nurse call system in inpatient ward

#### 4.2.1 Accomplishment

The identified opportunities for gamification were related to clear pre‐ and post‐operative goals and activity tracking. Clear, scheduled, progressive and personalized goals could be provided via interface of the developed digital patient journey solution to improve self‐management capabilities. To increase the patients' knowledge, motivation and accessibility in their new active role, pre‐ and post‐operative goals could be provided via a chronological and detailed checklist, which includes all required actions to be done by patients. The possibility to untick the checkbox should be provided. The following quote from the interviews illustrates awareness: ‘I could report how I'm progressing in returning to normal everyday life' (ID204, female with THA). Especially related to discharge goals, the following quote from the interviews illustrates a potential increase in motivation and the need for better accessibility: ‘Well yes it could give you enthusiasm and motivation. I've always noticed where the nurse puts a checker, but because I don't have glasses, I can't see clearly what it says out there’ (ID217, female with TKA).

Activity tracking could provide guidance and monitoring related to goals by providing notification and later a reminder. A diary with reminders as an activity‐tracking tool could support the patient by enabling reporting of daily exercise results. The following quote from the interviews illustrates this view: ‘I named the dates for the vertical columns, and I put in columns so wide that these repetitions could be written with a pen where you can see what has been done’. (ID210, male with TKA). The diary could enable a comparison between preoperative mobility against the personal target of postoperative mobility.

#### 4.2.2 Challenge

The identified opportunities for gamification were related to adaptive or increasing difficulty or upward tendency and personalized challenge. Preoperatively, adaptive or increasing difficulty or upward tendency in repetition and duration of exercises could be provided to support a patient's recovery from surgery and to prevent pain caused by the use of a walking aid, for instance. Post‐operatively, adaptive or increasing difficulty or upward tendency in repetition and duration of exercises could be provided to support a patient's progress in recovering, and activity tracking could provide information of progress. The following quote from the interviews illustrates this view: ‘At the beginning, however, we should go with small weights. How to lift that load, this application would be extremely good’ (ID220, female with THA).

Challenges must be personalized pre‐operatively based on a patient's background factors and the patient's living environment. Also, exercises must be personalized based on a patient's background factors, personal targets of post‐operative mobility and by taking into account the patient's living environment, whereas alternative exercises should be provided. The following quote from the interviews illustrates the need for alternative exercises: ‘It's just like this, and if you can't do this, then there is nothing’ (ID210, male with TKA).

#### 4.2.3 Guided

The identified opportunities for gamification were related to the quality of counselling as well as performance feedback and warnings. The content of counselling could provide information to the patient about health guarantee, health behaviour, preparation for surgery, events after arrival from home to the hospital (Leiko is from home to surgery unit in the hospital) before surgery, surgery itself, rehabilitation (in the inpatient ward and at home), traveling, medication, complications, digital joint certificate and contact information of service provider. Initial information, personal target of post‐operative mobility, previous surgeries, medications, dental examination, skin problems, homecare and other important personal information could be provided by the patient via information input and provision. Data from sensors used by patients could be provided to enable remote monitoring to be done by healthcare providers.

Implementation of counselling could be provided via methods like digital counselling material. The following quote from interviews illustrates the need for digital counselling material: ‘The rehabilitation instructions were at home, indeed so there, in the text of this referral, in my opinion, there was no mention that this should be included’ (ID210, male with TKA). Furthermore, methods such as remote visits with the help of a video call, a message system, a social networking platform, a feedback provider and a navigator could be provided. The following quote from interviews illustrates the need for a navigator: ‘The first time I went there, I never heard of a new Leiko like this before; what on earth is this Leiko?’ (ID212, female with THA).

Personalized counselling, with content that is meaningful for a patient with THA or TKA, could be provided to patients to address, for example, fears, certain lifestyle, personal needs, special needs, overall needs, surgery time during winter and distant municipality of residence. Patient‐centeredness could be improved by need‐based facilities, care, support and diet. The following quote from interviews illustrates this view: ‘Everyone should know what kind of patient comes and what the bed heights are’ (ID211, male with THA). The benefits of counselling could provide patient engagement related to lifestyle change and reaching clear goals. The following quote from interviews illustrates this view: ‘That you should now do this and this, and then the motivation will grow when there are clear instructions’ (ID220, female with THA). Performance feedback could be provided by patients by answering the query related to previous visits, for example, with the help of a mobile phone. Warnings could be sent to patients in needed cases.

#### 4.2.4 Playfulness

The identified opportunities for gamification were related to ease of use and visualization. A digital patient journey solution's ease of use could be addressed with the help of a simple and clear user interface and long‐lasting battery in sensors. A solution could visualize rehabilitation exercise results reported via diary by the patient, by drawing a curve and by showing status in relation towards a clear and personalized challenge goal. The remaining actions in relation to a clear goal and an estimate of the time needed to complete the remaining actions could also be visualized. The following quotes from interviews illustrate this view: ‘I think it's motivating, or so if you could still get the intelligence there. That if you've done this regularly, it would draw something like a curve… expected rehabilitation track or steps’ (ID220, female with THA).

Real‐time timespan visualization (in 1‐week accuracy) could increase the transparency of the entire arthroplasty journey, to give the patient a better possibility to plan and live their life pre‐ and post‐operatively and ensure that there are no gaps in information flow between different service providers. The following quotes from interviews illustrate this view:

‘Real‐time information about the whole thing’ (ID211, male with THA) and ‘It is precisely the predictability when it affects one's daily life and your own work and that of many others. It's easier to work things out if you know a little bit about what's coming’. (ID220, female with THA)

#### 4.2.5 Social experience

The identified opportunities for gamification were related to encouragement, monitoring and social networking. Social experience with peers, support networks and healthcare providers could provide patient encouragement related to fears, lifestyle changes, discharge and rehabilitation. This same solution could provide the possibility to monitor visualized feedback and reports. Patient accountability related to the remaining actions could be increased by providing the ability for patients to monitor the remaining actions in relation to reaching a clear goal. The following quote from interviews illustrates this view:

That you know where you're going. Whether you're behind on something, a goal, or an average… After all, I can quite easily influence the results of treatment, precisely through these exercises and adhering to them without compromise. (ID210, male with TKA)

A platform for social networking with peers, support networks and healthcare providers could be provided to support patients during the arthroplasty journey. In addition, it could enable direct communication between healthcare providers and patients during inpatient care. Peer support, however, should be supervised by healthcare providers.

## 5 DISCUSSION

This study addressed the gap in the existing research by identifying gamification opportunities for digital patient journey solutions to increase patients' engagement and motivation for health‐related behaviour in the arthroplasty journey. According to our knowledge, this is the first study to provide an important insight into the special needs of patients in terms of digital design and to highlight the active participation of patients in the co‐production of healthcare services in the chosen context.

Gamification opportunities were identified on five experiential dimensions defined by the framework of Högberg et al. (2019): accomplishment, challenge, guided, playfulness and social experience. Clear, scheduled, progressive and personalized goals, with an activity tracking, real‐time timespan visualization and social networking with peers, support networks and healthcare providers could be provided. Opportunities for competition and immersion were not identified.

In line with previous literature (Jansson, Koivisto, et al., 2020), the identified gamification opportunities regarding accomplishment were related to clear, scheduled, progressive and personalized goals, with activity tracking. According to our findings, clear goals and activity tracking could be provided prior to surgery in relation to the entire pre‐ and post‐operative phases to increase the patient's knowledge, motivation and accessibility related to the required actions to be done by the patient during the arthroplasty journey. A previous study based on healthcare providers' interviews also addressed that overall and partial goals need to be defined prior to surgery and easily understandable overall, and partial goals could be provided via a checklist of the required action points, with a scheduled checkbox to be ticked in an interface of the developed digital patient journey solution when actions are completed (Jansson, Koivisto, et al., 2020).

The identified gamification opportunities regarding challenges were related to the increasing difficulty or an upward tendency and personalized challenges. According to the findings, adaptive or increasing difficulty or upward tendency and personalized challenges regarding clear pre‐ and post‐operative goals could be provided to support safe and progressive rehabilitation in relation to personal target of postoperative mobility (finding ‘just‐right‐challenge’). It must be noted, however, that patients' personal targets related to post‐operative mobility are varying, whereas safe and personalized exercises could be provided to meet these targets. Prior research on task performance and creating the experience of accomplishment have indicated that the providing of clear and challenging goals increases the completion of provided tasks or reaching goals (Koivisto & Hamari, 2019; Locke & Latham, 2002).

The identified gamification opportunities regarding the experience of feeling guided were related to the quality of counselling as well as performance feedback and warnings. The experience of feeling guided in a timely manner by a digital patient journey solution could motivate patients to safely change their role from passive to active and increase their engagement related to clear goals. Prior research has indicated that well‐designed games are known to support intrinsic motivation because of the satisfaction of the basic psychological needs of autonomy, competence and relatedness (Johnson et al., 2016). However, according to the current study, digital guidance could encourage some patients and discourage others, which highlights the need for personalization possibilities when planning the guidance of patients.

The identified gamification opportunities regarding playfulness were related to ease of use and visualization. The experience of being able to monitor one's own real‐time status, throughout the entire arthroplasty journey, could enhance the integration of TKA/THA patient journey and the patient's personal and family life. Prior studies have found out that the new digital service enabled change in patient behaviour and patient activity, and information sharing is seen as an enabler of value creation and the associated benefits in the development of digital health service (Vieresjoki et al., 2021).

In line with Fernandes et al. (2021), the identified gamification opportunities regarding social support were related to encouragement, monitoring and social networking. Prior research has indicated that digitization of services plays a key role in the transformation of value creation into value co‐creation (Rantala & Karjaluoto, 2016). However, traditional episode‐based meetings, between the service provider and the patient, encourage some patients and discourage others, which highlights the need for personalization possibilities when planning interactions with the patient. Furthermore, as a new mode of interaction between the service provider and the patient's support network, social networking via a platform could be enabled for the support network (e.g. patient's family) to enhance their possibility to support the patient during the arthroplasty journey.

## 6 CONCLUSION AND RECOMMENDATIONS

Several opportunities for gamification were identified for digital patient journey solutions, which could be used in advanced care to increase patients' engagement and motivation for health‐related behaviour during the arthroplasty journey. The opportunities for gamification were identified related to supporting the experiential dimensions of accomplishment, challenge, being guided, playfulness and social experience.

Several opportunities for gamification were identified for digital patient journey solutions, which could be used in advanced care to increase patients' engagement and motivation to health‐related behaviour during the elective primary fast‐track total hip and knee arthroplasty patient journey. Unfortunately, however, the use of gameful solutions is not yet common, and research in the area is scarce. In general, gamification and gameful solutions could provide various possibilities for personalized counselling, remote monitoring and social support in order to increase the physical and cognitive performance, improve self‐care and self‐management capabilities (e.g. signs and symptoms), automatize data collection and enhance patient experience. In addition, exergames could be used to improve physical activity and thus, increase the quality of life in patients with THA/TKA.

The current study opens possible future avenues for exploring the use of gamification in the lower limb joint replacement journey. Various needs and limitations need to be considered when developing digital gamified solutions and more research into the effectiveness of such solutions will be required. Due to the contextuality of gameful solutions and their design, specific needs of different user groups and the consequential challenges in applying results from one context to another, further research in different healthcare settings is warranted.

## 7 LIMITATION

Limitations need to be considered when evaluating the findings of the study. First, this is a single‐centre study, and the results may not be transferable to dissimilar populations. Second, the questions were not pilot tested, because our study was a secondary analysis. In addition, opportunities for competition and immersion were not identified. Third, participants did not review the transcripts. However, because the transcripts were transcribed verbatim from the audio recordings, they can be considered reliable sources of information. Furthermore, the results of this study may not be transferable to patients with negative attitudes towards a digital patient journey solution. This limitation highlights the need for personalization possibilities when planning patient journeys. Despite the above‐mentioned limitations, the findings of this study provide important insights into the identified opportunities for gamification to support patients' engagement and motivation for health‐related behaviour through gameful experiences and suggests the biggest opportunities for gameful solutions.

## CONFLICT OF INTEREST

The authors have no conflict of interest.

## AUTHOR CONTRIBUTIONS

All authors conceived the idea and initiated the project. MJ collected the data. JJ and MJ performed the analysis. All authors participated in the interpretation of the results and critically reviewed the manuscript. All authors read and approved the final manuscript.

## ETHICS APPROVAL AND CONSENT TO PARTICIPATE

This study was approved by the relevant academic centre, and it was reviewed by the Ethics Committee of the Northern Ostrobothnia Hospital District, Oulu University Hospital, Oulu, Finland, during the autumn of 2018 (Decision No: 83/2018). The aim and the method of the study were explained to the participants, and they were also informed by a standard written information form. Written informed consent was obtained from participants prior to inclusion in the study to ensure that the participation was voluntary (World Medical Association Declaration of Helsinki 2013).

## CONSENT FOR PUBLICATION

Consent to publish was obtained from all participants who took part in this study.

## Supporting information

Appendix S1

## Data Availability

The data sets generated and analysed are not publicly available. Data sets are available from the authors on reasonable request and with permission from the relevant academic centre.
